# Diagnosing and Managing Paradoxical Deep Vein Thrombosis in a Patient With Congenital Afibrinogenemia

**DOI:** 10.1002/jha2.70328

**Published:** 2026-06-17

**Authors:** Altamash Jawadi, Malik Alqawasmi, Mahmoud Abdelsamia, Hemza Assed, Mohammad Adawee, Mohammad Badawy, Xuan Thuy T. Nguyen, Andres E. Mindiola Romero, Allison Burnett, Ala Ebaid

**Affiliations:** ^1^ Department of Internal Medicine The University of New Mexico Albuquerque New Mexico USA; ^2^ Division of Hematology/Oncology The University of New Mexico Cancer Comprehensive Center Albuquerque New Mexico USA; ^3^ School of Medicine The University of New Mexico Albuquerque New Mexico USA; ^4^ Department of Pathology The University of New Mexico Albuquerque New Mexico USA; ^5^ Antithrombosis Stewardship Pharmacy The University of New Mexico Albuquerque New Mexico USA

**Keywords:** congenital afibrinogenemia, deep vein thrombosis, D‐dimer, fibrinogen replacement therapy, inherited fibrinogen disorders

## Abstract

Congenital afibrinogenemia, classically a bleeding disorder, can paradoxically predispose to thrombosis, potentially due to disruptions in fibrin‐mediated thrombin clearance and plasmin activation. A young man with reported congenital afibrinogenemia presented with extensive deep vein thrombosis in the setting of undetectable fibrinogen and deceptively low D‐dimer, revealing the limitations of fibrin‐dependent assays. Management required carefully balanced anticoagulation alongside fibrinogen replacement with subsequent genetic confirmation of a homozygous *FGB* pathogenic variant. He completed 3 months of therapy with clinical improvement and residual chronic thrombotic changes, emphasizing the need for heightened vigilance and individualized anticoagulation strategies in this population.

## 1 Introduction

Congenital afibrinogenemia is a rare autosomal recessive disorder, defined by the complete absence of circulating fibrinogen, with an estimated prevalence of 1 in 1 million individuals [1, 2, 3, 4]. Fibrinogen is essential to hemostasis as the precursor of fibrin and a key mediator of platelet aggregation via binding to the platelet glycoprotein IIb/IIIa receptor [5]. Although afibrinogenemia is classically associated with bleeding due to failure of stable clot formation, paradoxical arterial and venous thrombotic events are increasingly recognized [1, 6]. Here, we report a patient with genetically confirmed afibrinogenemia who developed extensive deep vein thrombosis (DVT) successfully managed with carefully balanced anticoagulation and fibrinogen replacement.

## 2 Case Presentation

A Turkish male in his early 20s presented with 1 week of progressive left lower extremity pain and swelling following a soccer match. He reported a childhood diagnosis of congenital afibrinogenemia with prior bleeding episodes treated with fibrinogen replacement therapy. Laboratory evaluation (Table 1) revealed a normal complete blood count, markedly prolonged prothrombin time (PT), partial thromboplastin time (PTT), and thrombin time, undetectable fibrinogen by Clauss method, and a low D‐dimer. Fibrinogen activity level, fibrinogen antigen level, and fibrinogen antigen/activity ratio were all below the level of quantification, highly suggestive of congenital afibrinogenemia. Venous duplex ultrasound (VDUS) revealed extensive acute occlusive DVT extending from the left peroneal vein to the femoral profunda vein. Therapeutic anticoagulation was initiated with enoxaparin 1 mg/kg twice daily. Anti‐Xa monitoring was not performed, given standard weight‐based dosing and absence of renal impairment. After 2 days, he was transitioned to apixaban 5 mg twice daily due to the patient's religious restriction regarding porcine‐derived products. Concurrently, human fibrinogen concentrate (RiaSTAP) was administered with dosing guided by serial fibrinogen levels. Initial replacement targeted ≥ 50 mg/dL; however, given concurrent therapeutic anticoagulation and variability in measured levels, the target was increased to > 100 mg/dL to provide a more reliable hemostatic reserve. Initial dosing consisted of 1 vial (1026 mg), followed by escalation to 2 vials (2052 mg; approximately 29 mg/kg) after persistently undetectable fibrinogen levels. Subsequent dosing was adjusted in response to fluctuations in fibrinogen activity, with additional infusions administered as levels declined. Fibrinogen activity was monitored prior to subsequent infusions, with peak levels assessed approximately 1‐h post‐infusion. Dosing was typically administered every 2–3 days, with frequency adjusted based on trough levels. D‐dimer levels were not trended following fibrinogen replacement, given known limitations in this condition.

**TABLE 1:** Key laboratory findings at presentation.

Test	Result	Reference range
White blood cell (WBC)	11.0 × 10^3^/µL	4.0–11.0 × 10^3^/µL
Hemoglobin (Hgb)	15.5 g/dL	13.5–17.7 g/dL
Platelets	248 × 10^3^/µL	150–400 × 10^3^/µL
PT	> 200 s	8.9–14.6 s
aPTT	> 200 s	26–38 s
International Normalized Ratio (INR)	> 16.00	0.80–1.30
Thrombin time	> 200 s	< 17 s
Fibrinogen activity	< 35 mg/dL	170–450 mg/dL
Fibrinogen antigen	< 5 mg/dL	149–353 mg/dL
D‐dimer	< 215 ng/mL	0–500 ng/mL

The patient remained hospitalized for 14 days, during which he demonstrated clinical improvement defined by reduction in pain, improved ambulation, and decreased limb swelling, without bleeding complications. He was discharged on apixaban 5 mg twice daily with continued fibrinogen replacement using Fibryga, administered three times weekly with interval fibrinogen monitoring for a planned treatment duration of 3 months.

On follow‐up, genetic testing confirmed a homozygous pathogenic *FGB* variant. He completed 3 months of therapy with resolution of symptoms and no major adverse events. Repeat VDUS demonstrated chronic, non‐occlusive thrombotic changes. Long‐term fibrinogen prophylaxis was not initiated, given the absence of recurrent bleeding and the need to balance future thrombotic risk.

## 3 Discussion

Thrombotic complications in afibrinogenemia are increasingly recognized. In a review of 128 patients with inherited fibrinogen disorders, thrombotic events occurred frequently, both spontaneously and in association with triggers such as trauma, surgery, and pregnancy, highlighting that thrombotic risk is clinically meaningful and multifactorial [6]. In our patient, symptom onset after a soccer match suggests that even minor tissue injury and relative immobilization may be sufficient to tilt the hemostatic balance toward thrombosis in susceptible patients.

Several mechanisms have been proposed to explain this paradox. Persistent free thrombin activity due to absent fibrin‐mediated sequestration, impaired fibrinolysis, and von Willebrand factor‐mediated platelet activation may collectively promote thrombus formation despite profound abnormalities in clot‐based assays [1, 6, 7].

These mechanisms carry important diagnostic implications. In our patient, the low D‐dimer contrasted sharply with a significant thrombus burden, reflecting the absence of fibrin degradation products. Although widely used as a screening test for VTE with high negative predictive value in the general population, D‐dimer is unreliable in afibrinogenemia. Likewise, PT, PTT, and thrombin time are uniformly prolonged and do not correlate with thrombotic risk. Diagnosis of VTE, therefore, relies on clinical suspicion supported by imaging.

Management of thrombosis in afibrinogenemia is challenging, as anticoagulation must be balanced against inherent bleeding risk. In our case, LMWH was briefly initiated for its predictable pharmacokinetics and reversibility with concurrent fibrinogen replacement targeting fibrinogen activity levels > 100 mg/dL to provide a more reliable hemostatic reserve and mitigate bleeding risk in the setting of concurrent therapeutic anticoagulation. This individualized approach reflects the absence of standardized regimens for fibrinogen replacement in the setting of concurrent anticoagulation. Apixaban was selected for transition primarily due to the patient's religious restrictions, as well as for outpatient feasibility, acknowledging limited evidence but potential practicality in carefully selected patients. Monitoring anticoagulation remains challenging, as standard clot‐based assays are unreliable in the absence of fibrinogen; anti‐Xa levels may be considered for LMWH, but were not performed in this case.

Genetic testing plays a crucial role in confirming and classifying quantitative fibrinogen disorders, particularly when fibrinogen activity and antigen levels are undetectable or discordant [1]. It also informs inheritance patterns, which are essential for genetic counseling, especially in consanguineous families. For our patient, genetic testing confirmed his reported diagnosis (Table 2). His variant, c.862G>A, has a reported prevalence of 0.0026% in alleles in individuals of European (Non‐Finnish) descent in gnomAD [8]. This variant has been reported in the homozygous state in individuals with afibrinogenemia and in the heterozygous state in an individual with hypofibrinogenemia [9, 10, 11]. Although this *FGB* variant has been reported in afibrinogenemia, it has not been consistently associated with increased thrombotic risk, and genotype‐phenotype correlations remain poorly defined.

**TABLE 2:** Molecular genetic findings.

Genes analyzed: *FGA, FGB, FGG*				
Gene	Inheritance	Variant (zygosity)	gnomAD AF	Interpretation
*FGB*	AR, AD	c.862G>A (homozygous)	0.0026% European (non‐Finnish)	Likely pathogenic

Abbreviations: AD = autosomal dominant; AF = allele frequency; AR = autosomal recessive; gnomAD = Genome Aggregation Database.

Clinicians should maintain a high index of suspicion for thrombosis in afibrinogenemia, particularly in the presence of additional risk factors such as trauma or immobilization. Imaging is essential when thrombosis is suspected, as fibrin‐dependent assays will likely be misleading. This case provides additional anecdotal evidence supporting cautious use of apixaban with fibrinogen replacement and highlights the need for future prospective studies to better define thrombotic risk and guide the development of standardized, evidence‐based management strategies.

## Author Contributions

A.J. contributed to the Abstract, Introduction, Case Presentation, and Discussion, performed literature searches, created tables, and led manuscript formatting and editing. M. Alqawasmi obtained patient consent, initiated the case report, and drafted the manuscript, contributing to the Abstract, Introduction, Case Presentation, Discussion, and literature review. M. Abdelsamia identified the rarity of the case and provided editorial input. H.A. and M.B. contributed to table formatting. M. Adawee assisted with reference revision and submission. X.T.T.N., A.E.M.R., and A.E. contributed to manuscript editing, with targeted input on discussion, laboratory interpretation, and follow‐up outcomes, respectively. A.B. provided expert guidance on anticoagulation management. All authors contributed to the preparation and revision of this manuscript.

## Funding

The authors have nothing to report.

## Ethics Statement

The authors have nothing to report.

## Consent

Written informed consent was obtained from the patient for the publication.

## Conflicts of Interest

The authors declare no conflicts of interest.

## Data Availability

All data relevant to this case report are included in the article. Additional details are available from the corresponding author on reasonable request.
